# 947. Nocardiosis in Renal Transplant Recipients Linked to Decreased Utilization of Trimethoprim/Sulfamethoxazole During COVID-19

**DOI:** 10.1093/ofid/ofab466.1142

**Published:** 2021-12-04

**Authors:** Terrence McSweeney, Jennifer Marvin, Elizabeth A Cohen, Vincent Do, Kristen Belfield, Sarthak Virmani, Matthew Davis, Dayna McManus, Samad Tirmizi, Jeffrey E Topal

**Affiliations:** Yale New Haven Hospital, New Haven, CT

## Abstract

**Background:**

The renal transplant population is at increased risk of Nocardiosis due to impaired T-cell mediated immunity with immunosuppression. *Pneumocystis jirovecii* (PJP) prophylaxis with trimethoprim/sulfamethoxazole (TMP/SMX) provides coverage against *Nocardia* spp. unlike alternative agents such as atovaquone (ATQ), aerosolized pentamidine (AP), and dapsone. During the COVID-19 pandemic, patients receiving AP were transitioned to ATQ to avoid the use of nebulized medication. This, in turn, led to decreased use of TMP/SMX as patients on oral ATQ were not reassessed for the use of TMP/SMX as would have occurred while on AP. Additionally, an increased incidence of *Nocardia* infections was observed during this time. The objective of this study was to determine the association between the incidence of *Nocardia* infections and number of TMP/SMX prophylaxis-days in pre- versus COVID-19 cohorts.

**Methods:**

This was a single center retrospective chart review of all renal transplant recipients between September 2018 – August 2019 (pre-COVID-19 cohort) and April 2020 – March 2021 (COVID-19 cohort). Patients were included if they were at least 18 years of age and a recipient of a cadaveric or living donor kidney transplant. Exclusion criteria included multi-organ transplant, pediatric patients, and repeat transplants. The primary outcome was incidence of Nocardiosis within the first 6 months post-transplant in the pre- and COVID-19 cohorts.

**Results:**

A total of 218 patients were included (Table 1). Induction therapy and initial immunosuppression did not differ significantly between groups, nor did rates of rejection within 180 days of transplant (Table 2). Although the pre-COVID-19 cohort had a higher rate of neutropenia, there was no difference in median absolute lymphocyte count between the two groups. The COVID-19 cohort had a decreased percentage of TMP/SMX prophylaxis-days (59.2% vs. 72.5%, p < 0.0001) and an increased incidence of *Nocardia* infections in the first 6 months post-transplant (4% vs. 0%, p=0.0292). All 4 cases of *Nocardia* infections occurred in patients receiving ATQ.

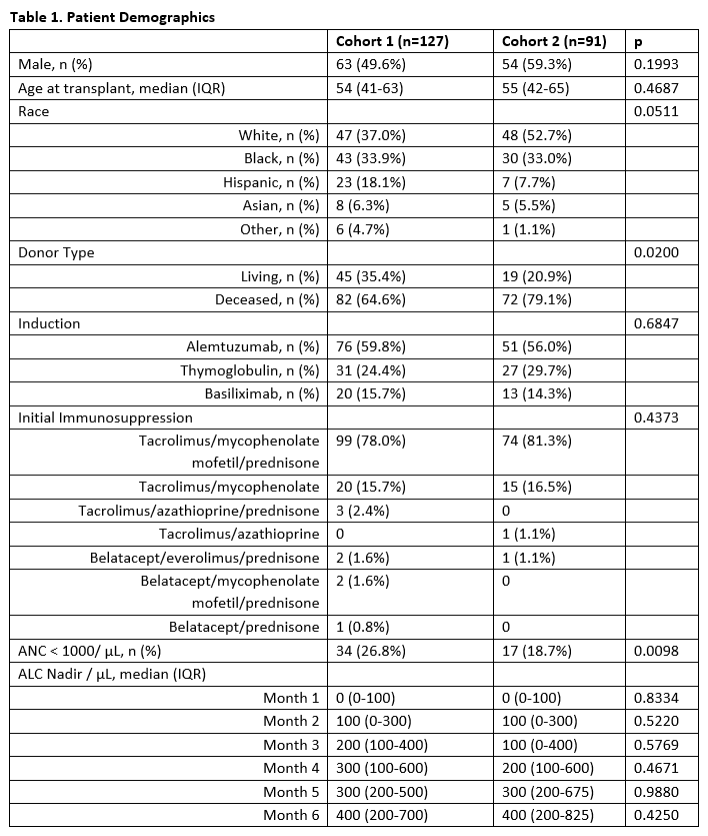

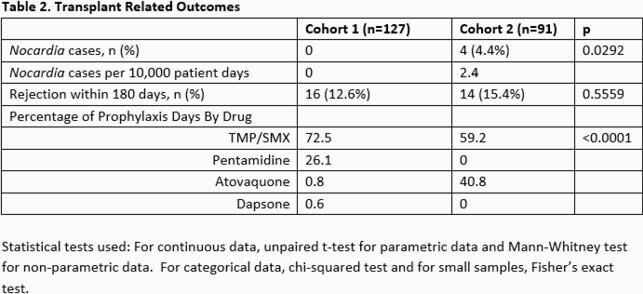

**Conclusion:**

The increased incidence of Nocardiosis was associated with a decreased use of TMP/SMX for PJP prophylaxis which may have been an unintended consequence of increased use of ATQ in lieu of AP during COVID-19.

**Disclosures:**

**All Authors**: No reported disclosures

